# First Detection of Spotted Fever Group Rickettsiae in *Ixodes ricinus* from Italy

**DOI:** 10.3201/eid0809.020060

**Published:** 2002-09

**Authors:** Tiziana Beninati, Nathan Lo, Hiroaki Noda, Fulvio Esposito, Annapaola Rizzoli, Guido Favia, Claudio Genchi

**Affiliations:** * Università degli Studi di Milano, Italy; † National Institute of Agrobiological Sciences, Tsukuba, Japan; ‡ Università di Camerino, Macerata, Italy; § Centro di Ecologia Alpina, Trento, Italy

**Keywords:** Rickettsia, *Ixodes ricinus*, Italy, tick-borne diseases, *gltA*, 17 kDa, *ompA*

## Abstract

*Ixodes ricinus* from Italy were examined for the first time to detect whether rickettsiae were present. Using molecular methods, we detected three different spotted fever group rickettsiae, including *Rickettsia helvetica*. Our results raise the possibility that bacteria other than *R. conorii* are involved in rickettsial diseases in Italy.

The genus *Rickettsia* comprises obligately intracellular, gram-negative bacteria. Before sequence-based classification methods were introduced, the genus was divided into two groups: the typhus group (TG), which included *R. prowazekii*, *R. typhi,* and *R. canada*, and the spotted fever group (SFG), which comprised all others. Recent phylogenetic studies of genes such as *gltA*, *ompA*, “gene D,” and that encoding the 17-kDa protein (hereafter referred to as “17kDa”) have shown that these two groupings are not consistent with species relationships; consequently, they have been modified (summarized in [1]). The TG now comprises only *R. prowazekii* and *R. typhi*, while the SFG contains seven divergent lineages: the *R. rickettsii* group, *R. japonica*, *R. montana*, the *R. massiliae* group, *R. helvetica*, *R. felis,* and the *R. akari* group. The AB bacterium, *R. bellii* and *R. canada,* cluster outside both the TG and SFG in most analyses (1).

Members of the SFG rickettsiae are usually associated with ixodid ticks, which transfer them to vertebrates via salivary secretions and between themselves transtadially and transovarially. Several tick-borne rickettsiae are causative agents of human or animal diseases. The prevalences of these diseases are primarily dependent on the geographic distribution of host ticks, which act as both vector and reservoir. Among rickettsiae found in Europe, *R. conorii* is probably the most well known. This bacterium, transmitted by *Rhipicephalus sanguineus*, causes “boutonneuse” or Mediterranean spotted fever (MSF), an endemic disease in several countries. Until recently, MSF was thought to be the only rickettsial disease prevalent in Europe, but in recent years some new human rickettsioses have been attributed to bacteria previously considered of unknown pathogenicity (2). An example is *Rickettsia helvetica*, which was originally isolated in 1979 from *Ixodes ricinus* but was shown to have pathologic relevance only in 1999, when it was associated with fatal perimyocarditis in two Swedish men (3). In addition, *R. helvetica* stimulated a specific antibody response in a man in France who had low-grade fever, headache, and myalgia (4).

In Italy, the only rickettsia isolated from humans and ticks thus far has been *R. conorii.* Since its host, *Rh. sanguineus,* favors warm climates, MSF is more common in central and southern Italy (5,6). In the years 1992–1998, approximately 8,500 cases of human rickettsioses presumed to be MSF were reported to the Italian Ministry of Health. Regarding the distribution of cases in different parts of Italy, some central (Lazio) and southern (Sardinia, Sicily, and Calabria) regions of the country have a particularly high morbidity rate, reaching an average of 11.9 cases for every 100,000 inhabitants in Sardinia, compared with the national average of 2.1.

The diagnosis of MSF in Italy usually depends on clinical evidence supported by serologic confirmation, mainly by the microimmunofluorescence (MIF) technique. A major limitation of MIF is cross-reactivity, which renders it unable to differentiate between various SFG rickettsiae (4). Thus, some cases of MSF in Italy, especially where the disease is not endemic, may in fact be due to other rickettsiae.

*I. ricinus* is found with high prevalence in the Italian Alps and Apennines (reaching 96% of all ticks collected in some areas) and in almost all other Italian regions that contain humid, forested habitats (7). While all life stages of *Rh. sanguineus* are mainly associated with dogs, *I. ricinus* can feed on >200 host species, primarily wild rodents and ruminants. In a survey in Liguria of ticks recovered from people, most ticks (89.3%) were *I. ricinus; Rh. sanguineus* was recorded less frequently (9.8%) (8).

To date, no studies have been conducted of potential rickettsiae in Italian ticks, other than *Rhipicephalus* spp. Recently, various *Rickettsia* species have been found in *I. ricinus* from other European countries, including *R. helvetica* in Switzerland, France, Sweden, Slovenia, and Portugal (4) and *Rickettsia* spp. IRS3/4 in Slovakia (9). To check whether such bacteria are also present in Italian *I. ricinus*, we studied specimens from three regions. We used molecular-sequence-based identification techniques, which offer high sensitivity and specificity compared with serologic tests and circumvent the need for bacterial culturing.

## The Study

**Figure 1:**
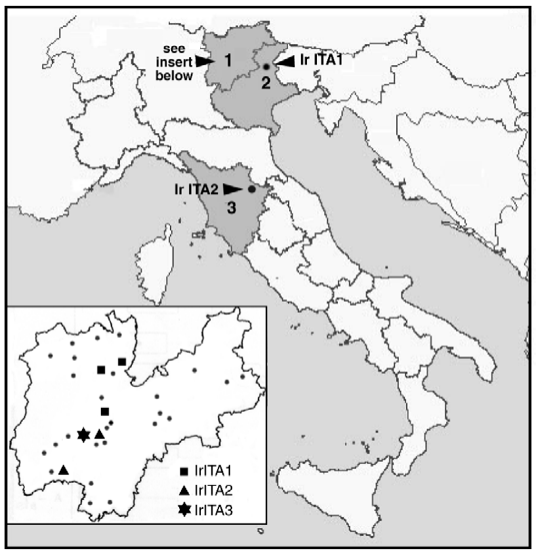
Location of *Ixodes ricinus* collection sites and detection of spotted fever group rickettsiae. 1). Trentino Province. Dots represent places where ticks were not found to have rickettsiae; different shapes represent the places where IrITA1 (Terlago, Denno, Vervó), IrITA2 (Molina di Ledro, Drena) and IrITA3 (Drena) were detected. In Feltre (2; Veneto Region) only IrITA1 was detected, while in Parco Nazionale delle Foreste Casentinesi (3; Toscana Region) only IrITA2 was detected.

A total of 109 *I. ricinus* specimens were collected in northern and central Italy (Figure 1), identified by using standard taxonomic keys, and stored at –20°C. Specifically, 89 ticks (70 adults and 19 nymphs) were collected by dragging vegetation in different parts of Trentino Province in April–October 1997 and 1999, and 10 ticks (7 adults and 3 nymphs) by dragging in Feltre (Veneto Region) in March 2000. Ten more ticks (7 adults and 3 nymphs) were collected from a patient at the Ospedale di Careggi in Firenze in May 1997. The man had been bitten in Parco Nazionale delle Foreste Casentinesi (Toscana Region; see Figure 1) a number of hours earlier but did not display any illness. MIF tests with *R. conorii* antigens were performed on his arrival at the hospital and again 4 weeks later; results were negative in each instance. Tick samples were placed in 50 μL of 10 mM Tris·HCl (pH 8.0), heated at 90°C for 10 min, crushed with a sterile plastic homogenizer, and treated with 10 μg of proteinase K at 50°C for 3 h. Polymerase chain reaction (PCR) of a 341-bp portion of *gltA* was performed by using the primers Rp CS.877p and Rp CS.1258n under conditions previously described (10). These primers were chosen for an initial screening because they are known to amplify all rickettsiae (11).

One hundred nine PCRs were performed, and nine positives (two adult females, three adult males, and four nymphs) were found. An initial estimate of the overall prevalence in Italian *I. ricinus* is thus 8.25%. To better establish intrageneric relationships, the nine positive samples were subjected to further PCR analysis with the primer pairs Rr 17.61p/Rr 17.492n and Rr 190.70p/Rr 190.602n (10), which amplified 394-bp and 488-bp portions of 17kDa and *ompA*, respectively. PCR bands for all three genes were then sequenced directly by using ABI PRISM sequencer (Perkin-Elmer, Foster City, CA). To compare the sequences obtained during this study with those of other rickettsiae, sequences present in GenBank were selected by means of BLAST as well as on the basis of previous reports (1,12). Sequences were converted to their putative amino acid sequences and aligned by using the program Clustal X (ftp://ftp-igbmc.u-strasbg.fr/pub/ClustalX/). Based on these alignments, nucleotide alignments were performed manually, and phylogenetic relationships were inferred by maximum likelihood (ML). The appropriate model of sequence evolution was determined by Modeltest 3.06 (http://zoology.byu.edu/crandall_lab/modeltest.htm), and trees were produced using the program TreePuzzle 5.0 (www.tree-puzzle.de), which provides branch lengths as well as quartet puzzling support values at each node with >50% support.

**Figure 2:**
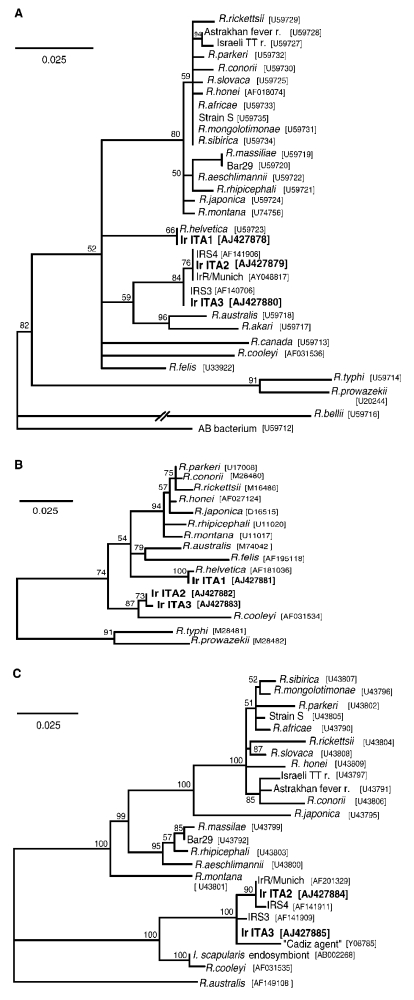
Phylogenetic analyses of rickettsiae based on *gltA* (A), 17 kDa (B) and *ompA* (C). Based on analyses in Modeltest 3.06, the models of substitution chosen for analysis in Treepuzzle 5.0 were TrN+G for *gltA* and 17kDa, and HKY+G for *ompA*. GenBank accession numbers for each sequence, including those found in this study (IrITA1-3), are shown adjacent to each strain. Numbers near each node represent quartet puzzling support values. Scale bars represent number of inferred substitutions at each site.

Comparisons of the sequences identified with those from closely related SFG *Rickettsia* spp. are shown in the Table; Figure 2 shows the results of phylogenetic analysis. *gltA*-based results (Figure 2a) show that all strains detected are SFG rickettsiae. For 17kDa (Figure 2b), no identical sequences for IrITA2 and IrITA3 were present in GenBank, and they clustered with *R. cooleyi* (isolated from *I. scapularis* in Texas [13]). *ompA* was the most variable of the three genes analyzed (Figure 2c) and could only be amplified from IrITA2 and IrITA3. Consistent with the results from *gltA*, *ompA* from IrITA2 was 100% identical to IrR/Munich; however, two substitutions were found between these two sequences and that of IRS4. Notably, for *ompA*, the cluster to which IrITA2 and IrITA3 belong also contains a strain detected in Spain (14). This finding suggests that these bacteria may be widespread in Europe. On the basis of *ompA* (and 17kDa) sequences, the clade containing IrITA2 and IrITA3 was closest to a clade containing *R.* *cooleyi* and an endosymbiont (10), both hosted by *I. scapularis*. All previous attempts to amplify *ompA* from *R. helvetica* by using various primers have failed, which suggests that the gene is either absent or too variable to work with primers designed from other SFG bacteria (12). This would explain why we were unable to amplify *ompA* from IrITA1. Taken together, the results from the three genes indicate that the clade containing IrITA2 and IrITA3 represents a lineage divergent from the seven described previously (1).

**Table:** Locations and numbers of ticks infected with spotted fever group (SFG) *Rickettsia* spp., and similarities between three genes in Italian SFG *Rickettsia* spp.^a^ with those from closely related bacteria from elsewhere in Europe^b^

			% Identity		
Sequence	Location (and no.) of infected *I. ricinus* specimens	SFG *Rickettsia* spp. with highly similar sequences present in GenBank	*gltA*	17 kDa	*ompA*
IrITA1	Trento Province (3) Feltre (2)	*R. helvetica*	100	99	–
IrITA2	Trento Province (2) Toscana (1)	IrR/Munich IRS4 (Slovakia)	100 100	– –	100 99
IrITA3	Trento Province (1)	IRS3 (Slovakia)	100	–	99

^a^The number of ticks examined from Trento, Feltre, and Toscana were 89, 10, and 10 respectively. ^b^*I., Ixodes*; *R., Rickettsia.*

## Conclusions

Our results represent the first demonstration of rickettsiae in Italian *I. ricinus* and the first use of molecular-sequence–based methods to identify rickettsiae in Italy. One bacterium, *R. helvetica,* occurs in several parts of Europe and has been implicated as a human pathogen. The other two strains have only recently been discovered in *I.* *ricinus* from Slovakia and Germany. Whether they are pathogenic is not known, but since other rickettsiae of previously unknown pathogenicity have subsequently been shown to be associated with disease (*R. helvetica* and *R. slovaca* [15]), these new strains warrant attention.

Several studies on rickettsioses in Italy have been published in the last two decades, and they all report *R. conorii* as the causative agent. As MSF is the only known rickettsiosis in Italy, diagnostic tests use *R. conorii* as the only antigen for serologic assays (16,17). However, since SFG rickettsiae cause cross-reactions, confusion about the source of the illness may occur. Although antibiotic therapy is generally effective for all SFG-related diseases, a better understanding of how different rickettsiae cause different symptoms will only come with their correct identification. During 1996–1999 in the regions we sampled, 23 rickettsioses (assumed to be MSF) were reported from Veneto, 42 from Toscana, and 3 from Trentino Province (Italian Ministry of Health, unpub. data). While many were likely to be MSF cases, the possibility exists that some were caused by other SFG (perhaps *R. helvetica*).

Unlike most studies, one serosurvey in northeastern Italy (18) used the complement-fixation test, which is less prone to cross-reactions (19); none of the sera tested was found positive for antibodies to rickettsiae. This finding may be explained by the use of *R. conorii*, *R. rickettsii*, *R. typhi,* and *R. akari* as the only antigens. Serosurveys such as these could therefore benefit from the use of antigens from the bacteria identified in our study. *I. ricinus* is one of the most abundant tick species in Italy, having a very low host specificity and a record of attaching to large numbers of humans (8). The results reported here add SFG rickettsiae to the list of potentially dangerous pathogens that Italian *I. ricinus* carry.
